# Highly improved accuracy of the revised PREoperative sarcoma score (rPRESS) in the decision of performing surgery for patients presenting with a uterine mass

**DOI:** 10.1186/s40064-015-1318-7

**Published:** 2015-09-17

**Authors:** Tomonori Nagai, Yasushi Takai, Taichi Akahori, Hiroaki Ishida, Tatsuya Hanaoka, Takahiro Uotani, Sho Sato, Shigetaka Matsunaga, Kazunori Baba, Hiroyuki Seki

**Affiliations:** Department of Obstetrics and Gynecology, Saitama Medical Center, Saitama Medical University, 1981 Kamoda, Kawagoe-shi, Saitama, Japan

**Keywords:** Uterine sarcoma, Preoperative diagnosis, Laparoscopic surgery

## Abstract

In 2014, we published an article titled “Novel uterine sarcoma preoperative diagnosis score predicts the need for surgery in patients presenting with a uterine mass” on the preoperative diagnosis of uterine sarcoma, in the SpringerPlus (Nagai et al. in SpringerPlus 2014, 3:678. doi:10.1186/2193-1801-3-678). Subsequently, we received several suggestions from readers, which were used to modify the statistical analysis methods and create a more precise preoperative diagnostic scoring system, which we present here as a supplemental report. The subjects were 63 patients who underwent surgical therapy for suspected uterine sarcoma (sarcoma group: 15 patients, benign group: 48 patients). Logistic regression analysis using the exact method was performed considering the subjects’ preoperative age, serum lactate dehydrogenase levels, magnetic resonance imaging findings, and endometrial cytology findings. We then used parameter estimates obtained from this analysis to revise the PREoperative Sarcoma Score (PRESS). The revised PRESS (rPRESS) has a maximum score of 10 points and an optimal cut-off value of 4 points, as derived from a receiver operating characteristic curve. Using this, the accuracy, positive predictive value, and negative predictive value were 93.7, 92.3, and 94.0 %, respectively. The diagnostic precision of the rPRESS is better than that of the original PRESS.

## Background

Uterine sarcoma is a non-epithelial malignant tumor that occurs in the uterus. It is resistant to chemotherapy and radiation therapy. The prognosis is extremely poor, except in cases in which early radical resection is possible (Gadducci et al. 2008). As the mass grows from the myometrium, approaching it from outside the body and making a pathological diagnosis from a biopsy can be difficult. Thus, the early diagnosis of uterine sarcoma often requires a hysterectomy and a pathological search. Preoperative diagnosis for uterine sarcoma using imaging and hematological findings has not exhibited a high accuracy. Therefore, many patients who undergo hysterectomy on a suspicion of uterine sarcoma are finally diagnosed with benign uterine myoma, the most common uterine mass. Only few patients are diagnosed with uterine sarcoma.

To improve the preoperative diagnosis of uterine sarcoma, we previously created and reported a preoperative diagnostic scoring system (called the PREoperative Sarcoma Score: PRESS) using several diagnostic predictive factors (Nagai et al. 2014). In the PRESS, we selected 4 diagnostic predictive factors from preoperative clinical, hematological, imaging, and endometrial cytology findings. Preoperative age, serum lactate dehydrogenase (LDH) levels, magnetic resonance imaging (MRI) findings, and endometrial cytology findings were used to create a preoperative diagnosis scoring system for uterine sarcoma. At its optimal cut-off value, the scoring system had an accuracy of 84.1 %, sensitivity of 0.8, and specificity of 0.854.

Readers commented on the article and suggested that the diagnostic accuracy of PRESS could be improved by modifying the statistical analysis method. They suggested that using the exact method instead of the maximum likelihood method with logistic regression analysis for the multivariate analysis would improve the accuracy of the analysis.

The maximum likelihood method is often used with parameter estimates in logistic regression analyses. However, when the occurrence probability is extremely small (or large), or if the number of samples is not enough for the number of parameters, it is sometimes impossible to obtain accurate estimates with this method. The exact method is one way of resolving these issues (Mehta and Patel 1995) (Heinze and Schemper 2002).

The present study used the subjects from our previous report (63 patients who underwent surgery based on a preoperative suspicion of uterine sarcoma). Four diagnostic predictive factors that exhibited significant differences in the univariate analyses (preoperative age, serum LDH level, MRI findings, and endometrial cytology findings) were used in multivariate analyses using a logistic regression analysis with the exact method. The results of these analyses were used to create the revised PRESS (rPRESS). We found that the rPRESS had a higher accuracy in the preoperative diagnosis of uterine sarcoma. Therefore, we present these findings in this supplemental report.

## Patients and methods

### Subjects

The subjects were the 63 patients from a previous report titled “Novel uterine sarcoma preoperative diagnosis score predicts the need for surgery in patients presenting with a uterine mass” (Nagai et al. 2014). These patients underwent surgery at the Saitama Medical Center of Saitama Medical University, between January 2006 and December 2012. Among them, 15 patients had uterine sarcoma and 48 patients had benign uterine tumor represented by uterine myoma (Table 1).

**Table 1:** Histological analysis

Pathological diagnosis		
Sarcoma (n = 15)		
Leiomyosarcoma	9	(60.0 %)
Adenosarcoma	3	(20.0 %)
ESS	3	(20.0 %)
Benign (n = 48)		
Cellular leiomyoma	4	(8.3 %)
Leiomyoma	42	(87.5 %)
Adenomyosis	2	(4.2 %)
Total	63	

This study complied with the guidelines specified in the Declaration of Helsinki and Japanese ethical guidelines for observational studies. Therefore, this study was approved by the Institutional Review Board (IRB) of Saitama Medical Center, Saitama Medical University, without the necessity to obtain written informed consent from the patients for the publication of this report.

### Statistical analysis

A logistic regression analysis using the exact method was performed on the predictive factors that exhibited significant differences in the univariate analyses preformed in our previous report (age, serum LDH levels, MRI findings, and endometrial cytology findings). The parameter estimates obtained from this were used to create the rPRESS. The cut-off values for the rPRESS were derived from a receiver operating characteristic (ROC) curve. *P* < 0.05 was considered statistically significant. The SAS system was used for the statistical analyses.

## Results and discussion

In our previous report (Nagai et al. 2014), univariate analyses revealed that age, serum LDH levels, MRI findings, and endometrial cytology findings were significant factors in the preoperative prediction of uterine sarcoma. The cut-off values for age and serum LDH levels were 49 years and 279 U/L, respectively. A positive MRI finding was, “intratumoral hyperintense signal on T1-weighted images and/or a heterogeneous signal on T2-weighted images.” For endometrial cytology classified using the Papanicolaou classification, a Class III or higher was considered as a positive finding. If Class III or higher was observed, an endometrial biopsy was performed. Patients who were diagnosed with a malignant tumor at this point were excluded from the present study. Table 2 shows the preoperative data for the predictive factors in the sarcoma and benign groups.

**Table 2:** Positive findings in patients

	Total	Sarcoma	Benign	*P* value (sar. vs. ben.)	Sensitivity	Specificity	PPV (%)	NPV (%)
Age (≥49 years)	31/63 (49.2 %)	14/15 (93.3 %)	17/48 (35.4 %)	<0.001*	0.93	0.65	45.2	96.9
Serum LDH level (≥279 U/L)	7/63 (11.1 %)	7/15 (46.7 %)	0/48 (0.0 %)	<0.001*	0.47	1.00	100.0	85.7
MRI findings	27/63 (42.9 %)	12/15 (80.0 %)	15/48 (31.3 %)	<0.001*	0.80	0.69	44.4	91.7
Cytological findings	6/63 (9.5 %)	5/15 (33.4 %)	1/48 (2.1 %)	<0.001*	0.33	0.93	83.3	58.3

* P < 0.05

In the present study, we performed a logistic regression analysis on these 4 factors, using the exact method, which found age, serum LDH levels, and endometrial cytology findings to be independent predictive factors (Table 3). We repeated the logistic regression analysis with the exact method on these 3 factors, to calculate parameter estimates (Table 4). We then created the rPRESS by referencing these parameter estimates. In the rPRESS, age ≥49 years is assigned 2 points, serum LDH level ≥279 U/L is assigned 4 points, and positive endometrial cytology is assigned 4 points, leading to a total of 10 points (Table 5). MRI findings were considered a scoring item in the original PRESS. However, as the multivariate analysis did not show them to be an independent predictive factor, they were eliminated from the revised version. Regarding point distribution, in the original PRESS, age, serum LDH levels, and endometrial cytology findings were assigned 2 points each, because they displayed significant differences in both the univariate and multivariate analyses, while MRI findings only received 1 point as they exhibited a significant difference only in the univariate analysis. In the rPRESS, the point distribution was based on the parameter estimates obtained from the logistic regression analysis using the exact method.

**Table 3:** Multivariate analysis of statistically significant positive findings

	Odds ratio	95 % CI	*p* value
Age	10.139	[1.639, ∞]	0.033*
Serum LDH level	36.988	[5.140, ∞]	<0.001*
MRI findings	0.498	[0.009, 7.114]	0.981
Cytological findings	42.527	[5.988, ∞]	<0.001*

* *P* < 0.05

**Table 4:** Parameter estimates of each predictor

	Odds ratio	95 % CI	*P* value	Parameter estimates
Age	9.159	[1.517, ∞]	0.039*	2.215
Serum LDH level	38.108	[6.215, ∞]	<0.001*	3.640
Cytological findings	41.040	[6.518, ∞]	<0.001*	3.715

* *P* < 0.05

**Table 5:** The revised PREoperative sarcoma scoring system (rPRESS)

Predictors	0 point	2 points	4 points
Age	<49	≥49	
Serum LDH level	<279		≥279
Cytological findings	Negative		Positive
Total	10 points		

**Fig. 1:**
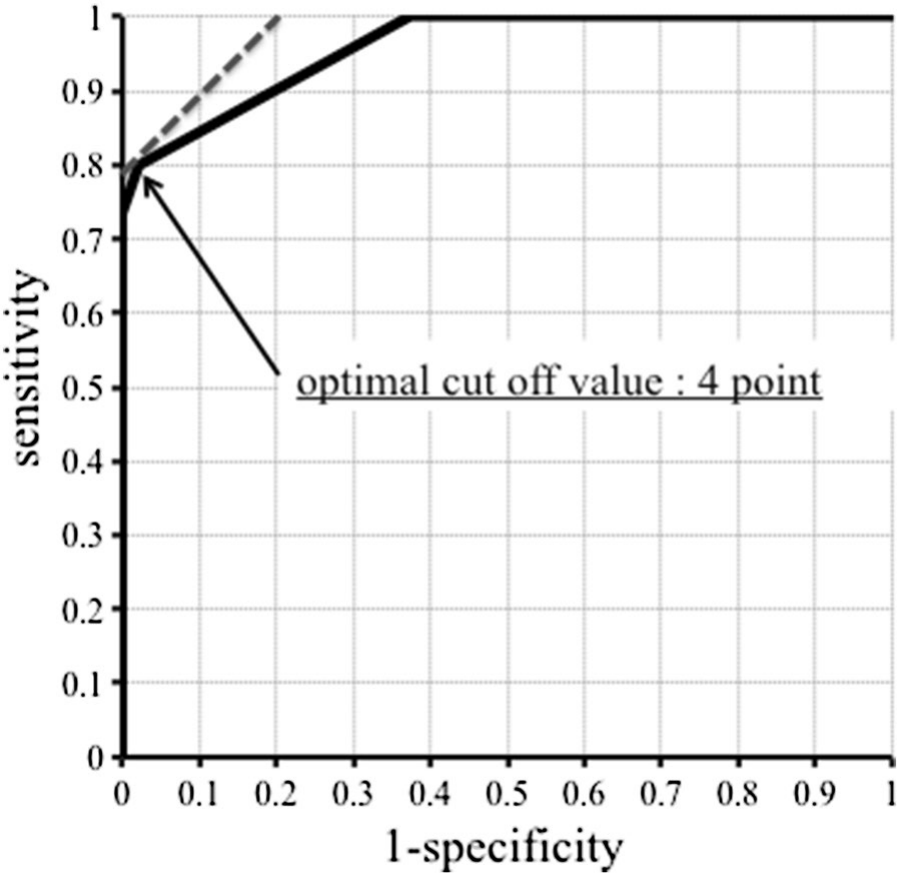
ROC curve for rPRESS. The optimum cut-off point based on the ROC curve analysis for the rPRESS was 4 points

With the rPRESS, mean scores of 5.067 and 0.792 were obtained in the sarcoma and benign groups, respectively, indicating a significant difference. With an ROC curve, the statistically optimal cut-off value for the rPRESS was 4 points (Fig. 1). When the score was 4 points or higher on the rPRESS, the accuracy, positive predictive value (PPV), negative predictive value (NPV), sensitivity, and specificity were 93.7, 92.3, 94.0 %, 0.8, and 0.979, respectively (Table 6).

**Table 6:** The revised PREoperative sarcoma score (rPRESS) and accuracy of rPRESS for all patients

Scoring (points)	0	2	4	6	8	10	Total	Mean score (range)
Sarcoma	0	3	1	11	0	0	15	5.067 (2–6)
Benign	30	17	1	0	0	0	48	0.792 (0–4)
Total	30	20	2	11	0	0	63	1.810 (0–6)
							*P* < 0.001 (sar. vs. ben.)	

Scoring (points)	≥0	≥2	≥4	≥6	≥8	≥10
Sarcoma	15	15	12	11	0	0
Benign	48	18	1	0	0	0
Total	63	33	13	11	0	0
Accuracy (%)	23.8	71.4	93.7	93.7	76.2	76.2
PPV (%)	23.8	45.5	92.3	100	–	–
NPV (%)	–	100	94.0	92.3	76.2	76.2
Sensitivity	1	1	0.8	0.733	0	0
Specificity	0	0.625	0.979	1	1	1

However, 3 cases with a rPRESS score of less than 4 points eventually received pathological diagnoses of uterine sarcoma (all scored 2 points). One of them was a 51-year-old post-menopausal woman who complained of lower abdominal pain. A 9-cm intrauterine tumor was found. The patient’s serum CA125 level was elevated (180 U/mL), but RI was low (0.39). No abnormalities were observed in serum LDH level, MRI findings, or endometrial cytology findings. The final pathological diagnosis after hysterectomy was ESS. The patient’s original PRESS score was also 2 points, and she was assessed to be negative. In addition, a 12-cm uterine tumor was found in a 71-year-old woman with abnormal vaginal bleeding. The intratumoral RI showed a low value (0.25), but the serum LDH level, MRI finding, and endometrial cytology finding showed no abnormality. After hysterectomy, the final pathological diagnosis was leiomyosarcoma. This patient’s original PRESS score was also 2, and she was assessed to be negative. The third patient was a 73-year-old woman with abnormal vaginal bleeding. She was found to have a 7-cm uterine tumor on MRI, suspected to be a uterine sarcoma. However, her serum CA125 level, serum LDH level, and endometrial cytology results were normal. The final postoperative pathological diagnosis was leiomyosarcoma. Her original PRESS score was 3, and owing to positive MRI findings, she was assessed to be positive. Using a cut-off value of 4 points would have missed these 3 cases. Considering that uterine sarcoma is a malignant tumor with poor prognosis, missed diagnoses are unacceptable. Therefore, our investigations suggest that surgery should be performed for a pathological diagnosis when the rPRESS score is 2 points or higher. This diagnostic score is ultimately only a reference in deciding the treatment plan, and the final decision on whether to operate should be made carefully for each individual case. In addition, considering the serum CA125 level, MRI findings, and intratumoral RI value, which were not used as predictive factors in the present study, further examination with a greater number of cases is necessary.

Recently, laparoscopic hysterectomy or myomectomy have become common forms of minimally invasive surgery for uterine myoma, which is a benign disease. However, the morcellation that occurs during such surgeries may accidently cause uterine sarcoma lesions to scatter in the abdominal cavity (Brower 2014) (Senapati et al. 2015). The rPRESS scoring system was created to act as a tool to select, as carefully as possible, cases in which uterine sarcoma is suspected before surgery. However, it may be useful as a preoperative screening tool to eliminate the possibility of performing laparoscopic surgeries intended to address what are thought to be benign uterine myoma, in cases where the disease is actually malignant. However, this scoring system was created as a tool for retrospectively examining data in particular cases in which uterine sarcoma is suspected before surgery. Therefore, it is not necessarily applicable as is for uterine sarcoma suspected to be benign uterine myoma. Consequently, further examinations are necessary to determine whether this scoring system is a valid screening tool when applied to multiple cases of uterine tumor thought to be benign uterine myoma before surgery.

## Conclusion

We found that the rPRESS had a better diagnostic accuracy and was more useful than the original PRESS. The rPRESS could help determine treatment plans for uterine masses suspected of being uterine sarcoma.

## Abbreviations

PRESS: Preoperative sarcoma score
rPRESS: Revised preoperative sarcoma score
MRI: Magnetic resonance imaging
LDH: Lactate dehydrogenase
IRB: Institutional review board
ROC: Receiver operating characteristic
ESS: Endometrial stromal sarcoma
PPV: Positive predictive value
NPV: Negative predictive value
